# Knockdown of circ-FURIN suppresses the proliferation and induces apoptosis of granular cells in polycystic ovary syndrome via miR-195-5p/BCL2 axis

**DOI:** 10.1186/s13048-021-00891-0

**Published:** 2021-11-16

**Authors:** Yongqian Chen, Jintian Miao, Ge Lou

**Affiliations:** 1 Department of Gynecology, The Affiliated Tumor Hospital of Harbin Medical University, No. 150, Haping Road, Nangang District, Harbin, 150081 China; 2 Department of Reproductive Medicine, The Sixth Affiliated Hospital of Harbin Medical University, Zhongyuan Avenue, Songbei District, Harbin, 150028 China; 3 Department of Gynecology, The First Affiliated Hospital of Harbin Medical University, No. 23, Youzheng Street, Nangang District, Harbin, 150001 China

**Keywords:** PCOS, Circ-FURIN, miR-195-5p, BCL2

## Abstract

**Background:**

Polycystic ovary syndrome (PCOS) is an endocrine disease that increases the risk of infertility. Circular RNAs (circRNAs) play important roles in regulating the biological processes of PCOS. Our study was designed to explore the function of circ-FURIN in PCOS.

**Methods:**

Circ-FURIN expression was detected using RT-qPCR. The protein expression of AVEN, BCL2, XIAP and AREL1 was measured using western blot. Dual-luciferase reporter and RNA pull-down assays were applied to clarify the interaction between miR-195-5p and circ-FURIN or BCL2. Functionally, cell proliferation was assessed by MTT and colony formation assays. Cell apoptosis was analyzed by flow cytometry.

**Results:**

Circ-FURIN was upregulated in PCOS patients and granular cells (GCs). Knockdown of circ-FURIN inhibited cell proliferation and promoted apoptosis of KGN cells, along with the increased expression of caspase-3 and Bax and the decreased levels of p-PI3K. Gene ontology (GO) analysis indicated circ-FURIN is associated with apoptotic signaling pathway and cell death. Subsequently, BCL2 expression was elevated in patients with PCOS and positively regulated by circ-FURIN. Furthermore, circ-FURIN was served as a sponge of miR-195-5p to directly target to BCL2. The levels of miR-195-5p were reduced in PCOS and KGN cells. Knockdown of circ-FURIN decreased the expression of BCL2, which was abolished by miR-195-5p inhibitor. At last, rescue experiments revealed that overexpression of BCL2 reversed the effects of circ-FURIN knockdown on cell proliferation and apoptosis of KGN cells.

**Conclusions:**

Loss of circ-FURIN alleviated the development of PCOS via miR-195-5p/BCL2 axis. Circ-FURIN may be the novel biomarker for PCOS.

## Introduction

Polycystic ovary syndrome (PCOS) is an endocrinopathy that causes anovulatory infertility in women at reproductive age. The clinical symptoms of PCOS are hirsutism, anovulation, and polycystic ovaries, which is characterized by hyperandrogenemia, hyperinsulinemia and disorders of cytokines [1, 2]. Globally, PCOS is popular among women at the age of 18–44 at the 5 to 15% incidence rates [3]. Moreover, PCOS is associated with pregnancy complications, such as gestational diabetes mellitus (GDM) and gestational hypertension [4]. However, due to various etiologies and overlapping symptoms, the diagnosis of PCOS is complex and delayed [5]. Furthermore, the side effects of current therapies for PCOS alleviates the clinical effects [6]. Therefore, to explore novel targets for diagnosis and treatment of PCOS is necessary.

Circular RNAs (circRNAs) are noncoding RNAs that act as sponges of microRNAs (miRNAs) by regulating protein function or controlling post-transcriptional gene expression [7, 8]. In mammalian cells, lots of circRNAs are abundant, conserved, and stable [9]. Numerous circRNAs are identified to exert their functions in the development stage and the states of physiology and pathology [10]. Abnormal expression of circRNAs is implicated with human diseases, including cardiovascular diseases, solid tumors, chronic inflammatory diseases, and neurological disorders [8]. circRNAs modulate many biological processes of PCOS, such as inflammation, proliferation, and endocrine [11]. Hsa_circ_0036881 (circ-FURIN), located in chromosome 15, is upregulated in PCOS [12]. However, its biological function is still unknown.

Thus, this study was designed to investigate the roles of circ-FURIN in PCOS and the molecular mechanism. Knockdown of circ-FURIN was found to suppress cell proliferation and induce apoptosis of KGN cells through regulating the miR-195-5p/BCL2 axis.

## Results

### Circ-FURIN was upregulated in PCOS

**Fig. 1:**
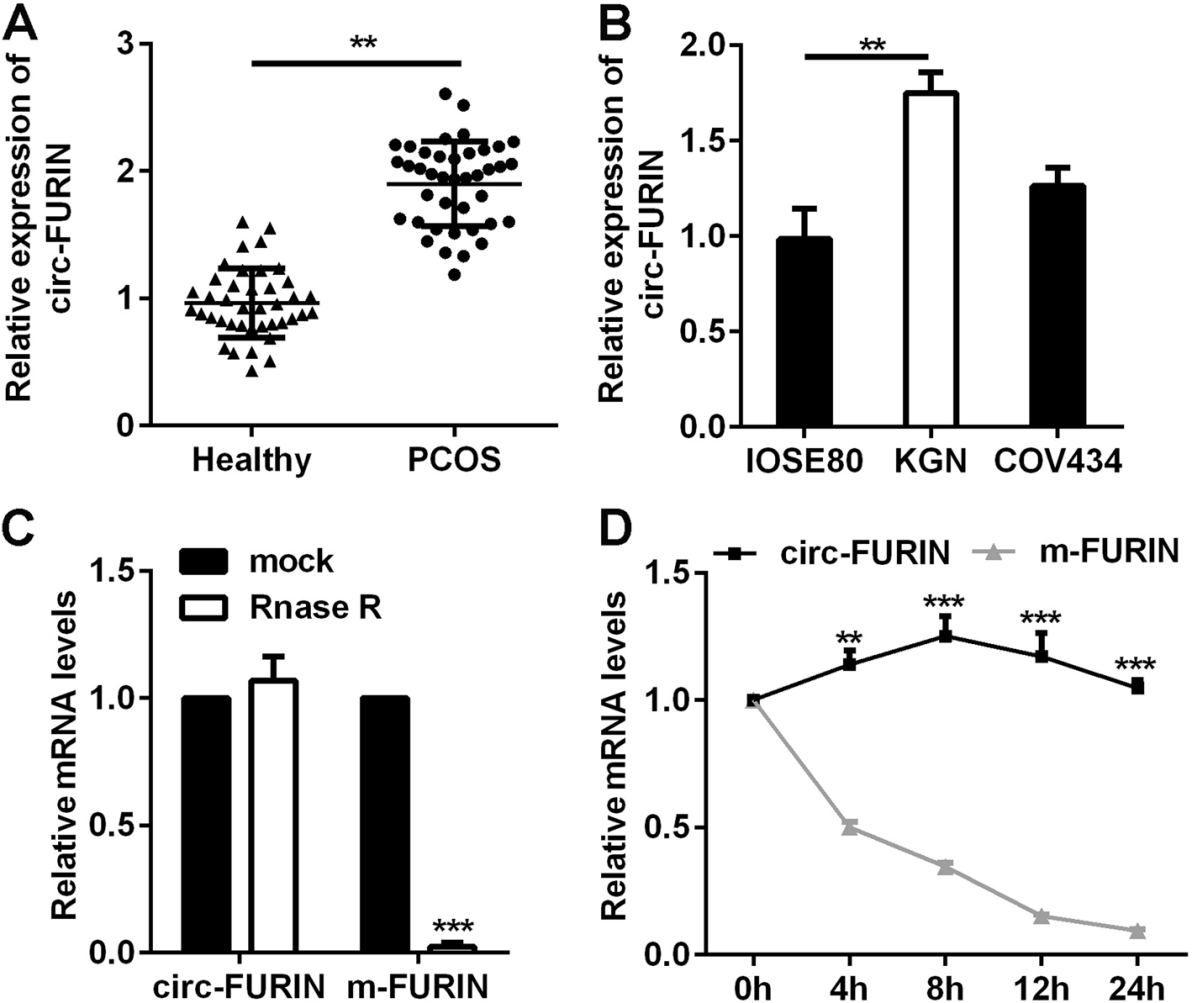
Expression of circ-FURIN was determined in PCOS. **A** Circ-FURIN was compared in CCs derived from patients with PCOS and healthy control using RT-qPCR. **B** Circ-FURIN was evaluated using RT-qPCR in IOSE80, KGN and COV434 cell lines. **C** The expression of circ-FURIN and m-FURIN was examined using RT-qPCR after RNase R treatment. **D** The expression of circ-FURIN and m-FURIN was assessed using RT-qPCR after Actinomycin D treatment. **P < 0.01. ***P < 0.001

Firstly, circ-FURIN levels in patients with PCOS and healthy control were tested using RT-qPCR. Circ-FURIN expression was significantly increased in cumulus cells (CCs) isolated from patients with PCOS, compared with healthy women (Fig. 1A). Similarly, circ-FURIN expression was significantly increased in KGN cells, compared to IOSE80 cells (Fig. 1B). Circ-FURIN levels were higher in COV434 cells than in IOSE80 cells, but there was no significant difference (Fig. 1B). Besides, the results of RNase R treatment showed that circ-FURIN was resistence to Rnase R, while FURIN mRNA was reduced (Fig. 1C). After Actinomycin D treatment, we found circ-FURIN was more stable than m-FURIN (Fig. 1D).

### Knockdown of circ-FURIN suppressed cell proliferation and facilitated apoptosis of KGN cells

**Fig. 2:**
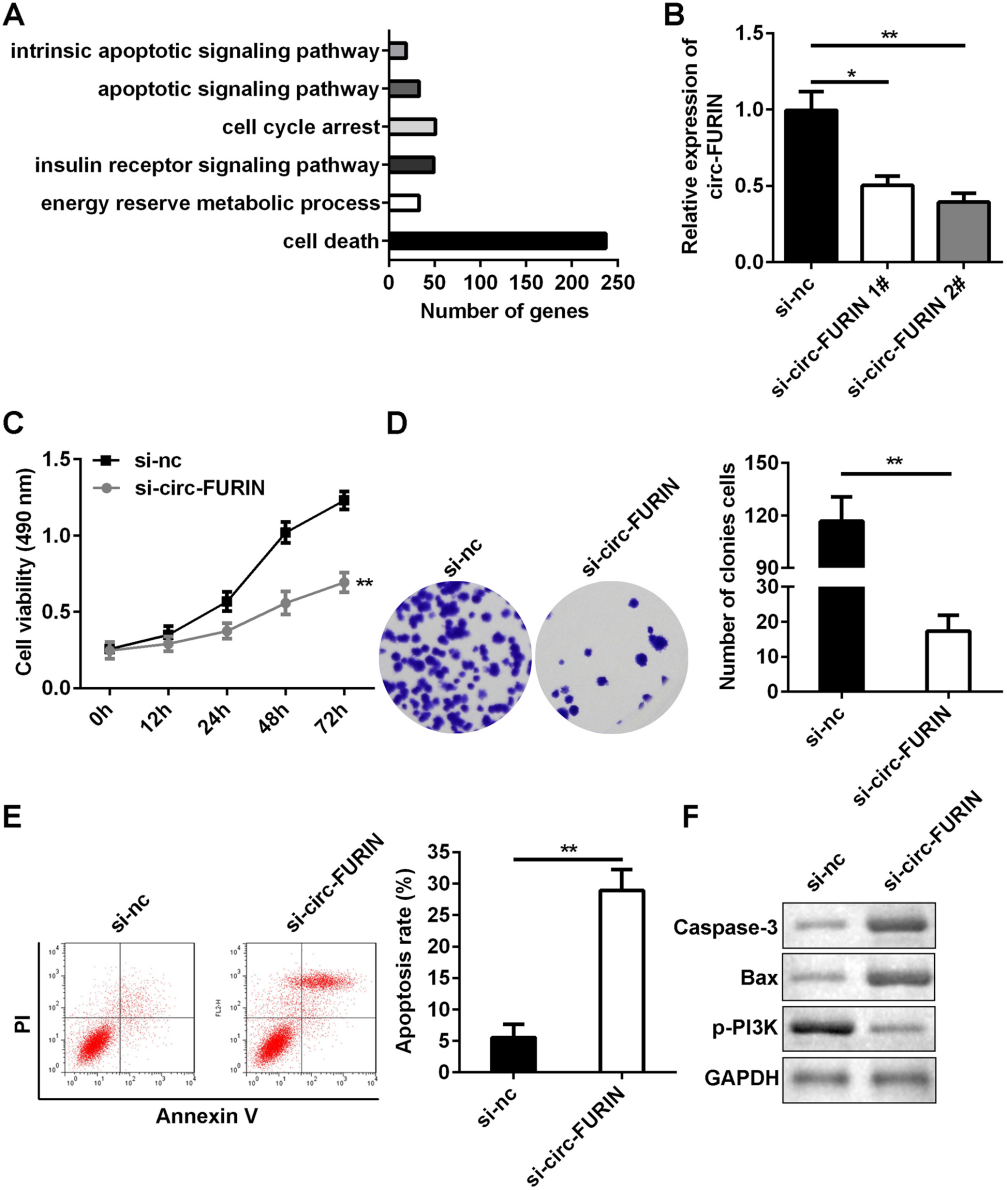
Effects of circ-FURIN knockdown on KGN cell proliferation and apoptosis. **A** The function associated with circ-FURIN were analyzed by GO analysis. **B** After transfection, circ-FURIN expression in KGN cells was tested using RT-qPCR. Post-transfection, **C** cell proliferation was assessed by MTT assay and **D** cell colony formation assay. **E** Cell apoptosis was determined using flow cytometry. **F** The protein expression of caspase-3, Bax and p-PI3K was conducted by western blot. *P < 0.05. **P < 0.01

According to Gene ontology (GO) analysis, we found circ-FURIN was associated with metabolic process, cell cycle, and cell death (Fig. 2A). Then, KGN cells were transfected with small interfering RNA (si)-circ-FURIN, and the results of RT-qPCR demonstrated that circ-FURIN was significantly reduced in si-circ-FURIN 1# (*P* < 0.05) and si-circ-FURIN 2# groups(*P* < 0.01) (Fig. 2B). si-circ-FURIN 2# was more efficient, which was used in the following experiments as si-circ-FURIN. Functionally, knockdown of circ-FURIN repressed cell viability and colony formation, compared with si-nc group (Fig. 2C and D). The apoptosis rates were significantly promoted by a loss of circ-FURIN (Fig. 2E). Furthermore, silence of circ-FURIN significantly increased the levels of caspase-3 and Bax but decreased p-PI3K levels (Fig. 2F).

#### BCL2 was elevated in PCOS

**Fig. 3:**
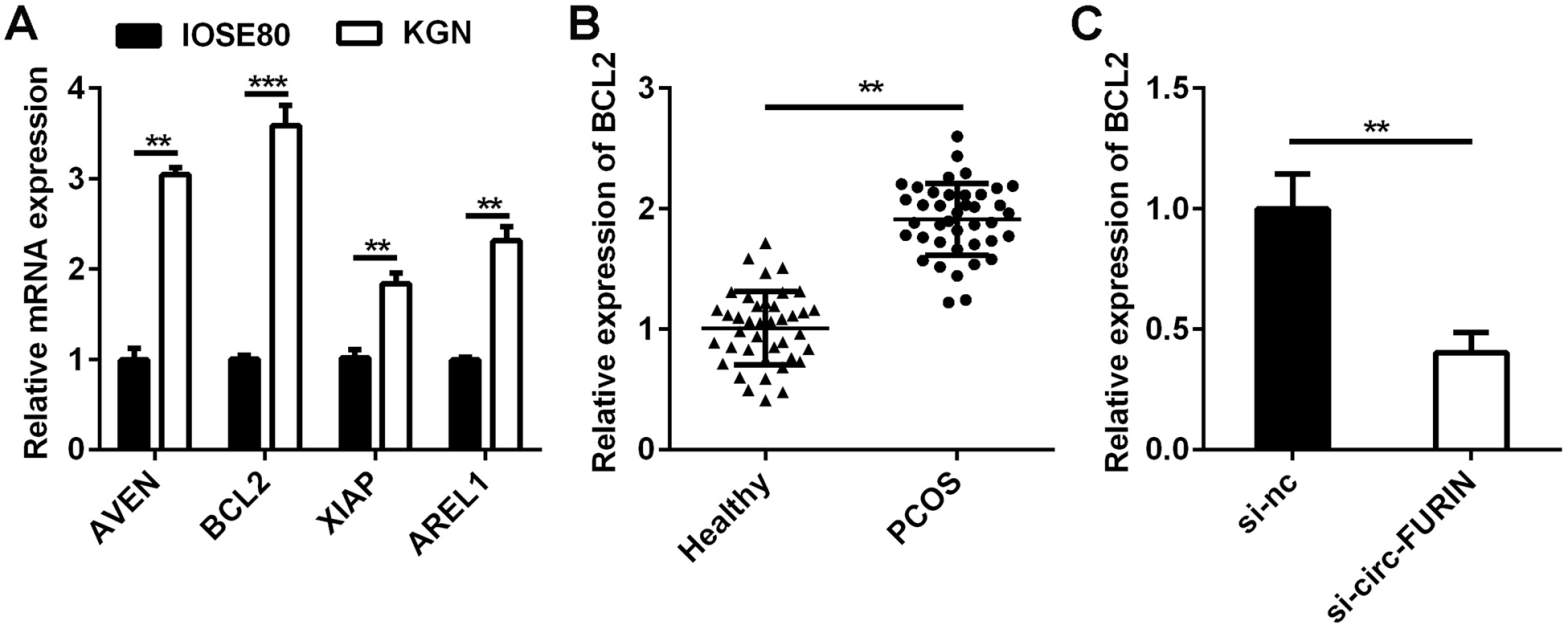
Expression of BCL2 was determined in PCOS. **A** RT-qPCR was conducted to test the expression of apoptosis-related factors (AVEN, BCL2, XIAP, and AREL1). **B** BCL2 expression was measured using RT-qPCR in GCs isolated from patients with PCOS and healthy control. **C** Transfection efficiency was examined using RT-qPCR in cells transfection of si-nc and si-circ-FURIN. **P < 0.01. ***P < 0.001

The levels of four apoptosis-related factors were detected in IOSE80 and KGN cells using RT-qPCR. The results showed that AVEN, BCL2, XIAP, and AREL1 levels were obviously higher in KGN cells than in IOSE80 cells (Fig. 3A). We chose BCL2 for further study. The levels of BCL-2 were also increased in patients with PCOS, compared with healthy control (Fig. 3B). Moreover, BCL2 expression was decreased by si-circ-FURIN in KGN cells, compared with si-nc (Fig. 3C).

#### Circ-FURIN upregulated the levels of BCL2 through competitively binding to miR-195-5p

**Fig. 4:**
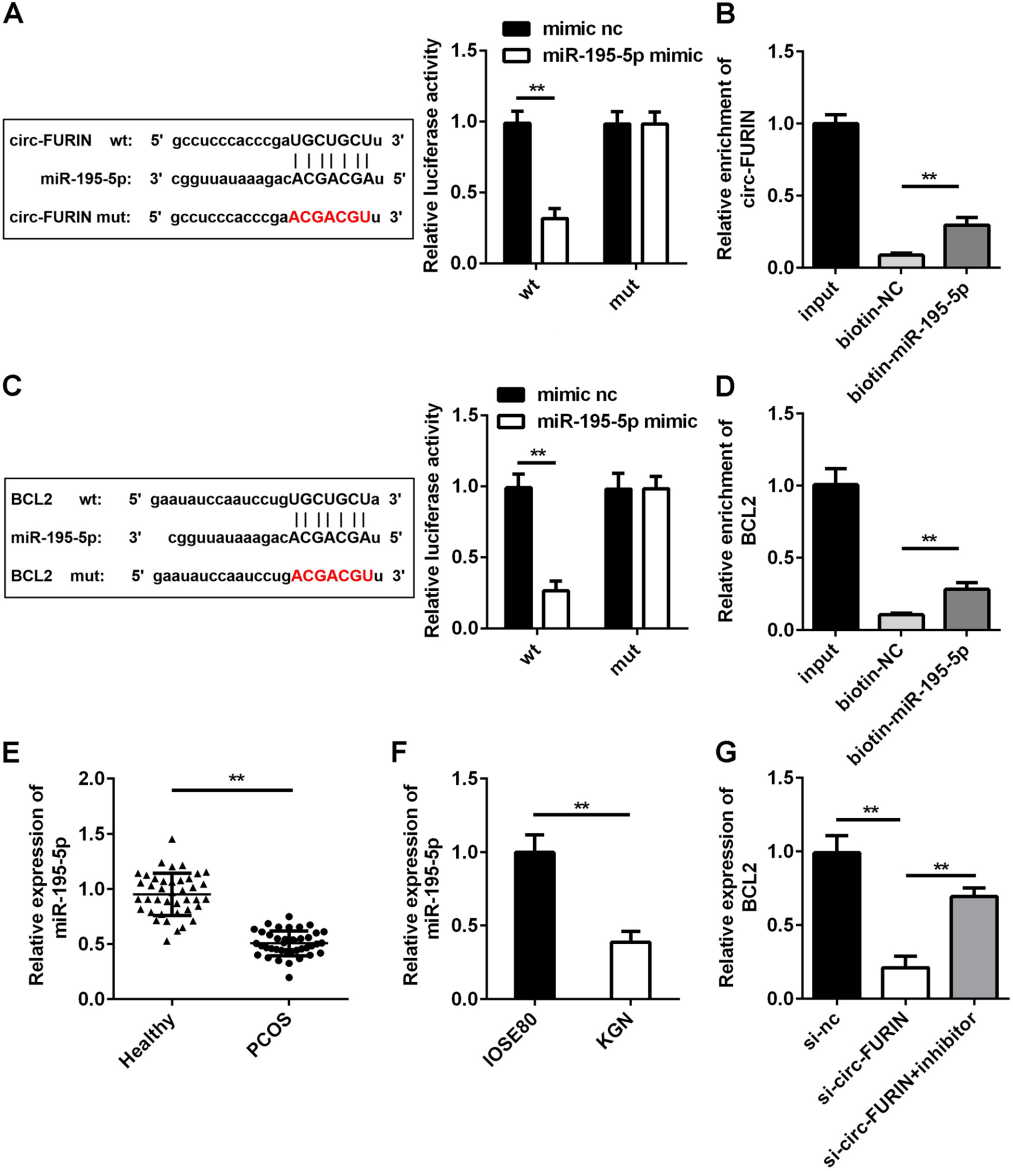
Circ-FURIN mediated BCL2 through sponging miR-195-5p. **A** The targeted relationship between circ-FURIN and miR-195-5p was predicted using bioinformatics and confirmed by dual-luciferase reporter assay. **B** RNA pull-down assay was utilized to confirm the binding of circ-FURIN to miR-195-5p. **C** The targeted relationship between miR-195-5p and BCL2 was predicted using bioinformatics and verified using dual-luciferase reporter assay. **D** RNA pull-down assay was utilized to confirm the binding of circ-FURIN to miR-195-5p. **E** MiR-195-5p was detected in GCs from patients with PCOS and healthy control using RT-qPCR. **F** MiR-195-5p was examined in IOSE80 and KGN cell lines using RT-qPCR. **G** The transfection efficiency was tested using RT-qPCR in cells transfection of si-circ-FURIN and si-circ-FURIN + miR-195-5p inhibitor. **P < 0.01

Bioinformatics data predicted the specific binding site of 3′ untranslated region (UTR) of circ-FURIN to miR-195-5p. Meanwhile, circ-FURIN wt and mut sequences were used for dual-luciferase reporter assay. MiR-195-5p mimic significantly reduced the luciferase activity in circ-FURIN wt group compared to mimic nc, while the difference in circ-FURIN mut groups show no significant changes (Fig. 4A). The results of RNA pull-down assay indicated that circ-FURIN expression was remarkably elevated in biotin labelled miR-195-5p group (Fig. 4B). On the other hand, the binding sites between miR-195-5p and BCL2 were predicted by bioinformatic analysis, and the targeted relationship was confirmed by dual-luciferase reporter analysis. Compared with mimic nc, the luciferase activity was significantly repressed by miR-195-5p mimic in BCL2 wt group, and no significant difference in the BCL2 mut group (Fig. 4C). RNA pull-down analysis further verified the interaction between miR-195-5p and BCL2 (Fig. 4D). Then, miR-195-5p was significantly downregulated in patients with PCOS and KGN cells (Fig. 4E and F). The expression of BCL2 was significantly decreased by knockdown of circ-FURIN, which was significantly abolished by miR-195-5p inhibitor (Fig. 4G).

### Overexpression of BCL2 promoted the proliferation and inhibited the apoptosis of KGN cells

**Fig. 5:**
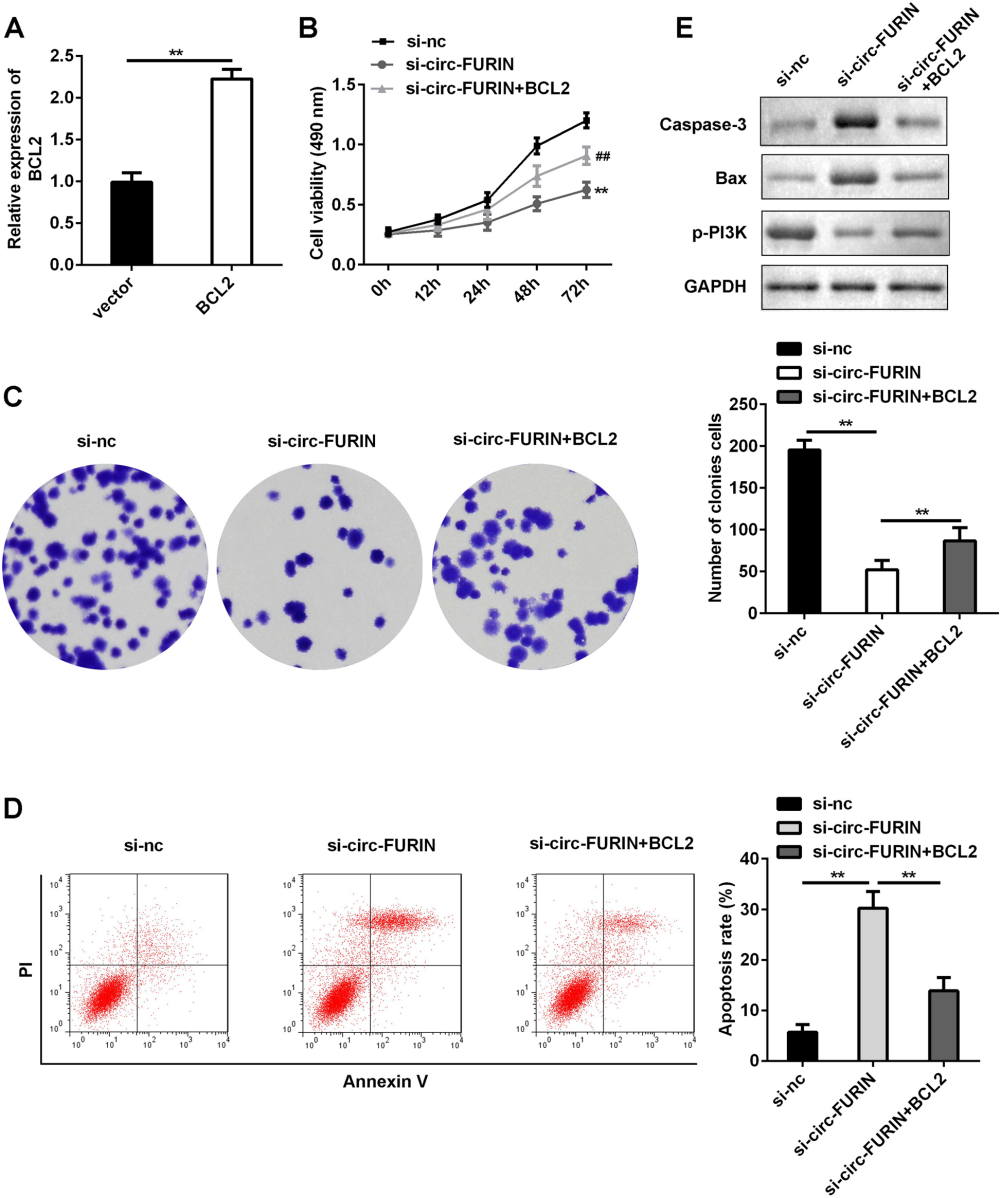
Effects of circ-FURIN and BCL2 on KGN cell proliferation and apoptosis. **A** The empty vector and BCL2 overexpressing vector were transfected into KGN cells, and RT-qPCR was conducted to examine BCL2 levels. Post-transfection, **B** cell proliferation was assessed by MTT assay and (**C**) cell colony formation assay (**D**) Flow cytometry was used to analyze cell apoptosis. **E** Caspase-3, Bax, and p-PI3K levels were conducted by western blot. ^##^*P* < 0.01. **P < 0.01

After transfection, BCL2 levels were elevated in BCL2 overexpressing group (Fig. 5A). Functionally, cell proliferation was inhibited by silence of circ-FURIN, which were abolished by overexpression of BCL2 (Fig. 5B and C). Overexpression of BCL2 reversed the facilitation of cell apoptosis induced by si-circ-FURIN w (Fig. 5D). Moreover, the regulatory effects of circ-FURIN knockdown on the protein expression of caspase-3, Bax and p-PI3K were reversed by overexpressed BCL2 (Fig. 5E).

## Discussion

Recent evidence has reported that circRNAs are associated with ovarian diseases, including PCOS [13], ovarian endometriosis [14], and epithelial ovarian cancer [15]. Herein, we identified the levels of circ-FURIN were elevated in patients with PCOS and KGN cells. Loss of circ-FURIN inhibited cell proliferation and facilitated apoptosis of KGN cells via miR-195-5p/BCL2 axis.

circRNAs have been revealed to be related to the pathological process of PCOS. For example, loss of circ_0118530 suppresses KGN cell proliferation, migration, oxidative stress, and inflammatory cytokine releases [16]. Decreased circLDLR in exosomes reduces estradiol production in PCOS [17]. Circ_0023942 is downregulated in PCOS, which inhibits the proliferation of granular cells [18]. Additionally, circ-FURIN (circ_0036877) acts as a novel biomarker for early pre-eclampsia [19]. According to the microarray assay, circ-FURIN (circ_0036881) expression is significantly upregulated in PCOS [12]. Herein, we also found the levels of circ-FURIN were increased in patients with PCOS as well as in KGN cells, which was consistent with the previous research [12]. Interesting. silence of circ-FURIN inhibited KGN cell proliferation and facilitated apoptosis of KGN cells. Meantime, caspase-3 and Bax levels were increased, and p-PI3K levels were decreased by knockdown of circ-FURIN. The data suggested that loss of circ-FURIN may attenuate the development of PCOS via degrading the cellular functions of KGN cells.

According to the results of GO analysis and enrichment analysis, circ-FURIN was associated with apoptotic signaling pathway and cell death via regulating apoptosis-related factors. We found BCL2 expression was the most remarkably overexpressed in KGN cells. BCL2, a member of the BCL2 family, has an anti-apoptotic effect in the intrinsic pathway of apoptosis [20, 21]. The abnormalities of BCL2 participate in the pathogenesis of numerous human diseases, including malignancies, neurodegenerative diseases, and autoimmune diseases [22–24]. In PCOS, BCL2 expression is increased in cumulus cells of mature oocytes, suggesting that BCL2 is related to the nuclear maturation of oocytes and fertilization [25]. In this study, BCL2 levels were upregulated in PCOS and was positively regulated by circ-FURIN. Moreover, overexpression of BCL2 abolished the effects of circ-FURIN knockdown on the proliferation and apoptosis of KGN cells. These findings suggested that loss of circ-FURIN affected cellular processes via regulating BCL2.

It is well known that circRNAs perform multiple functions via sponging miRNAs and RNA-binding proteins [26]. Our study found that miR-195-5p could directly bind with circ-FURIN, and BCL2, suggesting that circ-FURIN or BCL2 have targeted relationship with miR-195-5p. Most of the research on miR-195-5p has focused on cancers (such as cervical cancer, glioma, and lung cancer) [27–29], as well as endocrine disorders (such as gestational diabetes mellitus, cardiomyocyte hypertrophy, and acute kidney injury) [30–32]. However, few studies have shown the function of miR-195-5p in PCOS. In the present study, the expression of miR-195-5p was decreased in PCOS and KGN cells. Circ-FURIN increased BCL2 expression via sponging miR-195-5p. These results suggested that loss of circ-FURIN alleviated the progression of PCOS probably through miR-195-5p/BCL2 axis.

## Conclusions

Circ-FURIN was upregulated in PCOS and GCs. Loss of circ-FURIN suppressed cell proliferation and facilitated apoptosis of KGN cells. The mechanism is associated with the miR-195-5p/BCL2 axis. This study revealed that circ-FURIN might be a therapeutic target of PCOS.

## Methods

### Participants

Eighty participants (40 PCOS and 40 healthy control) were diagnosed with or without PCOS in the Affiliated Tumor Hospital of Harbin Medical University according to the Rotterdam standard. All participants had no history of drug allergy or other diseases. They provided written informed consent prior to this study. The study was approved by the Affiliated Tumor Hospital of Harbin Medical University.

### CCs collection

After 36 h of HCG administration, cumulus-oocyte complexes (COC) were extracted by vaginal puncture under a transvaginal ultrasound probe. CCs were obtained by mechanical stripping from COC. After washing with PBS, CCs were collected and stored at − 80 °C for further RT-qPCR analysis.

### Cell culture

KGN, COV434 and IOSE80 cells were purchased from the Chinese Academy of Science (Shanghai, China). KGN cells were cultured in DMEM/F12 supplemented with 10% FBS and 1% penicillin-streptomycin (All purchased from Gibco, ThermoFisher Scientific). IOSE80 cells were cultured in DMEM supplemented with 10% FBS and 1% penicillin-streptomycin. The cell culture conditions were 37 °C with 5% CO_2_.

### Cell transfection

si-circ-FURIN (si-circ-FURIN 1#: AACCAGTGTGCGAGGAAGGCT, si-circ-FURIN 2#: ACCAGTGTGCGAGGAAGGCTT), si-negative control (nc; UUCUCCGAACGUGUCACGUTT), miR-195-5p inhibitor (GCCAAUAUUUCUGUGCUGCUA) and negative control inhibitor (CAGUACUUUUGUGUAGUACAA), miR-195-5p mimic (UAGCAGCACAGAAAUAUUGGC) and negative control mimic (UUCUCCGAACGUGUCACGUTT), empty vector, and BCL2 overexpression vector were obtained from GenePharm (Shanghai, China). Lipofectamine 3000 (Invitrogen, Carlsbad, CA, USA) was used for transient transfection following the manufacturer’s instrument. Forty-eight hours later, transfection efficiency was examined using RT-qPCR.

### MTT assay

Cell proliferation was examined using MTT (Sigma-Aldrich, St. Louis, MO, USA) assay. At 48-h post-transfection, cells were incubated at 37 °C for 0, 12, 24, 48 and 72 h. Twenty microliter, five milligram per milliliter MTT was added into plates and continued to culture for 4 h. After washing with PBS, cells were treated with 150 μl dimethyl sulfoxide (Sigma-Aldrich). The OD value was determined by a microplate reader at 490 nm.

### Colony formation assay

Cells were seeded in 6-well plates (1 × 10^3^ cells/well) and incubated for 14 d at 37 °C. Then, the cells were washed with PBS, fixed with 4% formaldehyde, and stained with 0.1% crystal violet. The colony number was counted using five random fields.

### Cell apoptosis assay

Cell apoptosis was assessed using Annexin V-FITC Apoptosis Detection Kit (Sigma-Aldrich). Briefly, cells were resuspended in binding buffer at the density of 1 × 10^6^ cells/ml post-transfection. Five microliter Annexin-V FITC and 10 μl Propidium Iodide (PI) were added into 500 μl cell suspension and incubated at room temperature for 10 min in shade. The apoptosis of cells was tested using a flow cytometer.

### RNase R assay

Total RNA was isolated from CCs derived from patients with PCOS using Trizol reagent (Invitrogen). Two point five microgram total RNA was incubated with 10 U Rnase R (Geneseed, Guangzhou, China) at 37 °C for 30 min. RT-qPCR was determined after inactivation of Rnase R (70 °C for 10 min).

### Actinomycin D assay

After the cells incubation for 24 h, cells were treated with 1 μg/ml actinomycin D (Abcam) at 37 °C for 0, 4, 8, 12 and 24 h. Relative expression of circ-FURIN and m-FURIN was evaluated by RT-qPCR.

### RT-qPCR

Total RNA was isolated using TRIzol reagent (Invitrogen). The concentration of RNA was tested by spectrophotometry. RT-qPCR were determined using Hifair III One-Step RT-qPCR SYBR Green Kit (Yeasen, Shanghai, China). The reaction condition of RT was 42 °C for 10 min. QPCR was performed on ABI 7700 real-time PCR system (Applied Biosystems) with the following conditions: 95 °C for 5 min, followed by 40 cycles of 95 °C for 10 s and 60 °C for 30 s. The relative expression (fold change) was calculated using the 2^−ΔΔCT^ method. GAPDH and U6 were the endogenous controls. The specific primer sequences are shown in Table 1.

**Table 1:** Primer sequences for qPCR

Name	Forward (5′-3′)	Reverse (5′-3′)
Circ-FURIN	CGTGCAGACTATGCAAACCAG	TTCTCGGTGCTATAGTGCGT
AVEN	GACTTCAGTGTCCTCTTGAG	CCTTGCCATCATCGTTTCTC
BCL2	GGAAGGTAGTGTGTGTGG	ACTCCACTCTCTGGGTTCTTGG
XIAP	GACAGTATGCAAGATGAGTCAAGTCA	GCAAAGCTTCTCCTCTTGCAG
AREL1	GAGGGGACCGGACTATTTATGA	TCCTTCCAATCCCAGGAGACT
miR-195-5p	CGCAGCACAGAAATATTGGC	CTCAACTGGTGTCGTGGAGTC
GAPDH	GGAGCGAGATCCCTCCAAAAT	GGCTGTTGTCATACTTCTCATGG
U6	GCTTCGGCAGCACATATACTAAAAT	GCTTCGGCAGCACATATACTAAAAT

### Western blot

Total protein was extracted using RIPA lysis buffer (Yeasen), and BCA Protein Quantification Kit (Yeasen) was used to measure the protein concentration. Thirty microgramof protein was separated by 10% SDS-PAGE, followed by transferred onto PVDF membranes. Then primary antibodies including anti-caspase-3 (ab13847, 1:500), anti-Bax (ab53154, 1:1000), and anti-p-PI3K (ab32089, 1:1000) and anti-GAPDH (ab9485, 1:2500) were applied to incubate the membranes at 4 °C overnight. Then the membranes were incubated with goat anti-rabbit IgG (ab205718, 1:5000) for 2 h at room temperature. All antibodies were acquired from Abcam (Cambridge, MA, USA). Enhanced ECL Chemiluminescent Substrate Kit (Yeasen) was conducted to expose each band.

### GO analysis

The potential roles of circ-FURIN were revealed by GO analysis using DIANA-mirPath v.3 online tool (http://snf-515788.vm.okeanos.grnet.gr/) [33].

### Dual-luciferase reporter assay

The partial 3′ UTR region of circ-FURIN (sense: 5′-AAACTAGCGGCCGCTAGTGCCTCCCACCCGATGCTGCTTT-3′; antisense: 3′-TTTGATCGCCGGCGATCACGGAGGGTGGGCTACGACGAAA-5′) and BCL2 (sense: 5′-AAACTAGCGGCCGCTAGTGAATATCCAATCCTGTGCTGCTAT-3′; antisense: 3′-TTTGATCGCCGGCGATCACTTATAGGTTAGGACACGACGATA-5′) containing miR-195-5p binding sites were inserted into the downstream of firefly luciferase site of pmir-GLO vectors (Promega, Madison, WI, USA) using T4 DNA ligase to construct recombinant plasmids (circ-FURIN-wt and BCL2-wt). Mutation circ-FURIN (sense: 5′-AAACTA GCGGCCGCTAGTGCCTCCCACCCGAACGACGATT-3′; antisense: 3′-TTTGATCGCCGGCGATCACGGAGGGTGGGCTACGACGAAA-5′) and BCL2 (5′-AAACTAGCGGCCGCTAGTGAATATCCAATCCTGACGACGAAT-3′; antisense: 3′-TTTGATCGCCGGCGATCACTTATAGGTTAGGACACGACGATA-5′) sequences were synthesized using Mut Express II Fast Mutagenesis Kit (Vazyme, Nanjing, China). Similarly, circ-FURIN-mut and BCL2-mut plasmids were also inserted into pmir-GLO vectors. KGN cells were co-transfected with wt or mut recombinant plasmids and mimic nc or miR-195-5p mimic (Genepharm) using Lipofectamine 2000 (Invitrogen). Forty-eight hours post-transfection, the luciferase activity was tested using Luciferase Reporter Assay Substrate Kit (Abcam). Renilla activity was the endogenous control.

### RNA pull-down analysis

KGN cells were transfected with biotin-labeled miR-195-5p and NC for 48 h, respectively. Then the cells were incubated with lysis buffer for 10 min following washed with PBS. The lysate was incubated with Dynabeads M280 streptavidin (Invitrogen) for 3 h at 4 °C. The enrichment of circ-FURIN and BCL2 was evaluated using RT-qPCR.

### Data analysis

All data in this study were analyzed using GraphPad Prism 6.0 (La Jolla, CA, USA) from at least three repeated experiments. Statistical comparisons were determined using Student’s t-test and one-way ANOVA. The results were represented as mean ± standard deviation (SD). *P* < 0.05 dictated statistical significance.

## Data Availability

No data were used to support this study.

## Abbreviations

CC: Cumulus cell
circRNA: Circular RNAs
GC: Granular cell
GO: Gene ontology
miRNA: microRNA
mut: Mutant
PCOS: Polycystic ovary syndrome
RT-qPCR: Real-time quantitative polymerase chain reaction
siRNA: Small interfering RNA
UTR: Untranslated region
wt: Wile-type
